# The Under-representation of Canadian Women in Gastroenterology from Residency to Leadership

**DOI:** 10.1093/jcag/gwab020

**Published:** 2021-07-23

**Authors:** Noor Jawaid, Jordan LoMonaco, Natasha Bollegala

**Affiliations:** 1 Gastroenterology Residency Training Program, Department of Medicine, University of Toronto, Toronto, Ontario, Canada; 2 Division of Gastroenterology, Department of Medicine, University of Toronto, Toronto, Ontario, Canada; 3 Division of Gastroenterology, Department of Medicine, Women’s College Hospital, Toronto, Ontario, Canada

## Abstract

**Background:**

To determine representation of women in gastroenterology (GI) at residency and leadership levels in Canada.

**Methods:**

The Canadian Resident Matching Service provided data for internal medicine (IM), general surgery (GS), GI and cardiology applicant cycles 2014 to 2018. *Z*-tests were used to compare proportion of women entering each residency program. An internet search was conducted to calculate percentages of women as GI association presidents, residency program directors, division heads and oral speakers at conferences.

**Results:**

IM residency had on average of 1789 applicants with 487 matched (49.4% versus 49.5% women). GS residency had on average 357 applicants with 90 matched (41% versus 54.4% women). GI residency had on average 46 applicants with 34 matched (37% versus 35.3% women). Cardiology residency had on average 76 applicants with 54 matched (29% versus 27.8% women).

The Canadian Association of Gastroenterology (CAG) has had two out of 47 (4.2%) women presidents. The Ontario Association of Gastroenterology (OAG) has had no women presidents (0/9). The Association des gastro-entérologues du Québec (AGEQ) has had two out of 15 (13%) women presidents. The Alberta Society of Gastroenterology (ASG) has had one out of five (20%) women presidents. From 2018 to 2020, university division heads ranged from 0% to 13.3% women (0 to 2/15). University GI training program directors ranged from 28.6% to 35.7% (4 to 5/14). Women speakers at CAG’s annual conference varied 27% to 42% from 2016 to 2020, averaging 32.7%. Women speakers at OAG’s, AGEQ’s and ASG’s annual conferences averaged 23.3%, 24.1% and 35%, respectively.

**Conclusion:**

Women gastroenterologists display low representation at multiple levels along the GI career pathway.

## Introduction

Representation of women in the field of gastroenterology has remained a challenge. Graduating medical school classes in Canada are now 50% or more female but this percentage diminishes as medical trainees approach the GI residency match (1,2). Although the number of practicing gastroenterologists in Canada has been on the rise in the last 20 years, female representation has stayed low at 31% (3). There is no clear consensus on why this is the case or where along the line of training (medical school, internal medicine residency or GI residency) this phenomenon has its roots. Many theories have been postulated in the literature such as the ‘glass ceiling’ referring to an invisible barrier to advancement embedded in institutional culture and the ‘leaky pipeline’ referring to the loss of females along the training pathway due to work–life misbalance (4). It is also unclear whether there is something intrinsic to GI that causes a drop-off in women trainees. Low representation of women diminishes further along the GI career path with many examples in the literature including female senior authorship over the past 20 years (5).

The majority of research in the area of gender diversity has been conducted in the United States but there are two Canadian studies of note. Heathcote et al.’s 1997 survey-based study explored gender diversity among Canadian gastroenterologists in order to better understand what barriers to advancement existed for women in the field and why. The study concluded that professional success could be attained by either gender equally but it required disproportionate personal sacrifices on the part of women gastroenterologists (6). More recently, Perera et al. found that female Canadian gastroenterologists continued to report more difficulty with career advancement and attaining work–life balance than their male counterparts (7).

The main purpose of this study was to determine the proportion of women’s representation in GI at the residency level compared to other similar specialties and to document gender representation in major GI leadership roles in Canada. Our hypothesis was that with an increasing societal focus on gender proportion and increasing rates of gender balance in medical school, that proportionate gender representation in GI had improved compared to previous assessments.

## METHODS

The Canadian Resident Matching Service (CaRMS) is the national platform through which medical students apply for Canadian residency positions across all specialties. Both applicants and residency training programs provide their preferred rank to CaRMS which utilizes a predetermined algorithm to then ‘match’ applicants to residency positions which are contractually binding. CaRMS’ direct inquiry contact for research purposes was utilized to attain annual trend data for residency matches. The service allowed a maximum of 5 years’ worth of aggregate data to be divulged in order to protect the anonymity of applicants. General internal medicine (GIM) and general surgery (GS) statistics were analyzed for the post-graduate year (PGY) 1 match. GS was chosen as the comparator group due to the similar length of residency, on-call and work hours. Cardiology was chosen as the comparator to a GI residency training program in the PGY4 match because it also has a procedural training component and similar on-call and work hours to GI. It should be noted however that in Canada, cardiology differs from GI in that it is a 3-year residency training program versus a 2-year residency training program.

Official websites from Canadian GI associations and universities were used to gather data on gender representation among leadership positions. These included presidential history for national and provincial GI associations, university affiliated GI training program directors and academic university GI division heads. The Ontario Association of Gastroenterology (OAG) and Canadian Association of Gastroenterology (CAG) websites were used to gather archival data on oral speakers at national conferences (October 2019). The Alberta Society of Gastroenterology (ASG) and the Association des gastro-entérologues du Québec (AGEQ) provided annual conference programs directly via email request as the information was not available in full on their respective websites. These oral sessions included expert talks as well as presentations of key abstracts on the given sub-topic. Organizations were contacted directly for any missing historical data.

### Gender Classification

Gender was classified into men or women following a systematic assessment firstly, of given name. If the name alone did not make gender clear, an internet search of the individual’s given name was used to elicit captioned pictures and/or gender-specific descriptors including pronouns from academic websites or publicly available social media websites. A combination of these elements was then used to classify gender.

### Statistical Analyses

*Z*-tests were used to compare proportion of women entering each residency program. A *P*-value of <0.05 was considered significant. If any data were missing from within the minimum allotted 5-year time frame (2015 to 2020), every effort was made to contact the relevant organization or university program to acquire a complete set of data.

### Research Ethics

This study was exempt from institutional ethics review board approval because all of the data analyzed were publicly available.

## RESULTS

**Figure 1.**
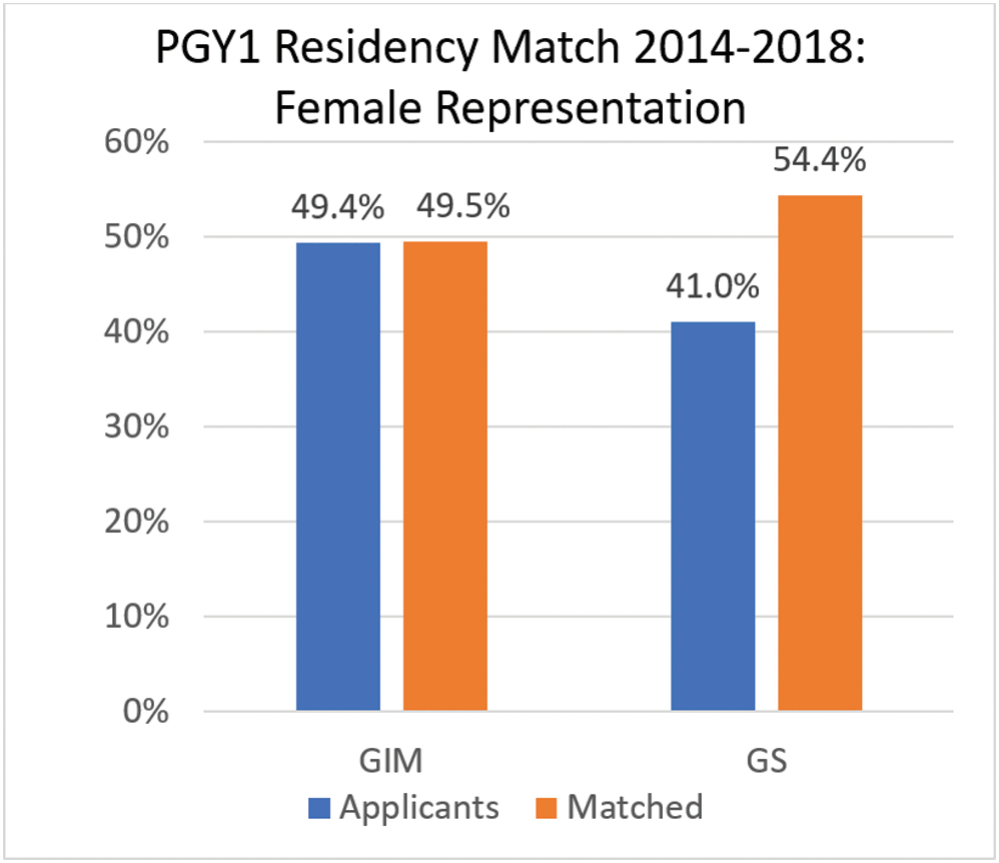
Comparing female applicants vs. females matched in the CaRMS PGY1 residency match by specialty 2014–2018. GIM, general internal medicine; GS, general surgery.

CaRMS data were used in two separate analyses: PGY1 and PGY4 matches from 2014 to 2018. The PGY1 match compared entry into IM and GS residency programs. In IM, the average number of annual applicants was 1789 (49.4% women) and the average number of matched applicants was 487 (49.5% women). In GS, the average number of annual applicants was 357 (41% women) and the average number of matched applicants was 90 (54.4% women) (Figure 1). In total, 742 women applied to GS from 2014 to 2018 with 244 matching (33%). For IM over the same period, 4420 women applied with 1206 matching (27%). Women applying to IM were less likely to match successfully compared to women applying to general surgery (27% versus 33%; *z* = −2.82, *P* < 0.01). Furthermore, compared to men, there was still a significantly lower proportion of women who matched to GIM than GS (*z* = −2.58; *P* < 0.01).

**Figure 2.**
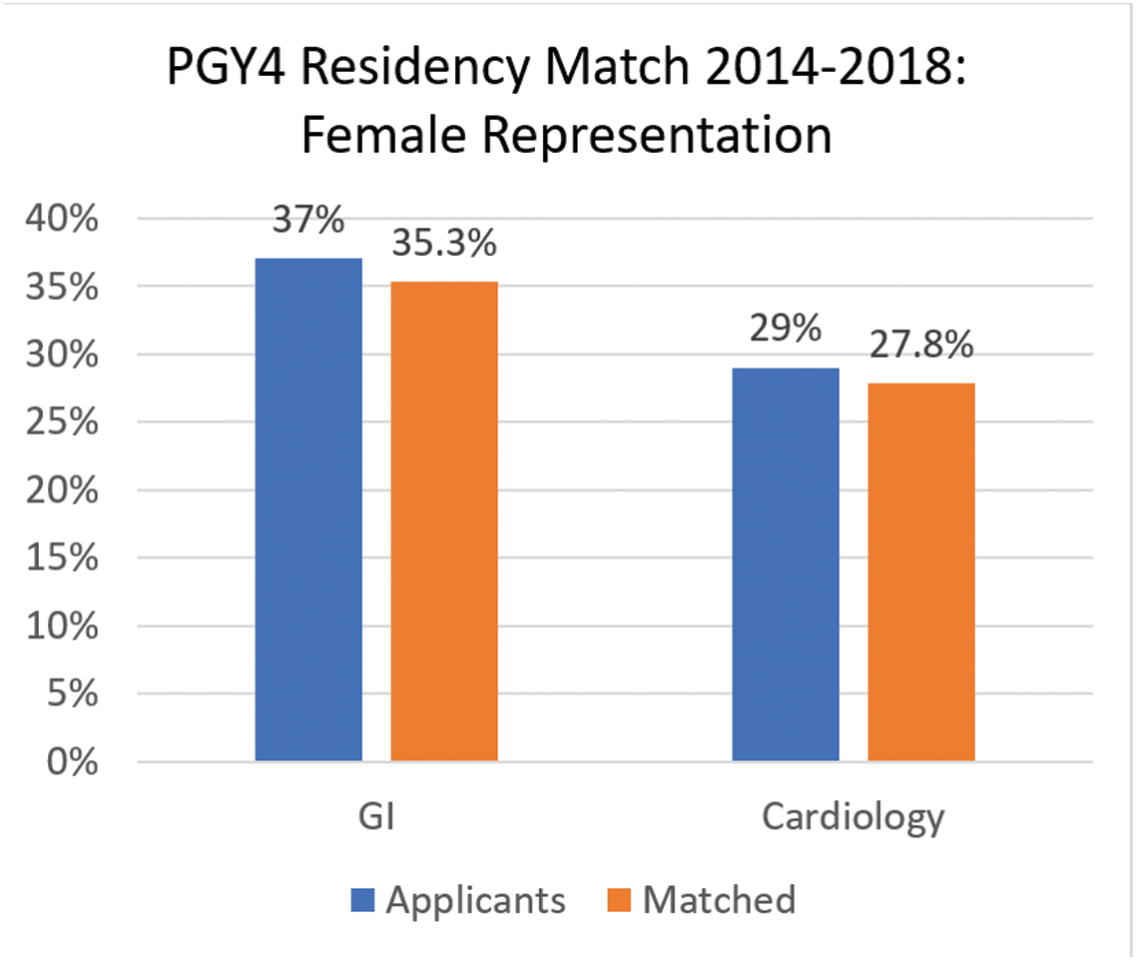
Comparing female applicants vs. females matched in the CaRMS PGY4 residency match by specialty 2014–2018. GI, gastroenterology.

The PGY4 match compared entry into GI and cardiology residency programs. In GI, the average number of applicants annually was 46 (37% women) and the average number of matched applicants was 34 (35.3% women). In cardiology, the average number of annual applicants was 76 (29% women) and the average number of matched applicants was 54 (27.8% women) (Figure 2). Women matched at an identical rate of 71% to both GI and cardiology. There was no significant difference in the proportion of women compared to men matching to GI versus cardiology.

**Figure 3.**
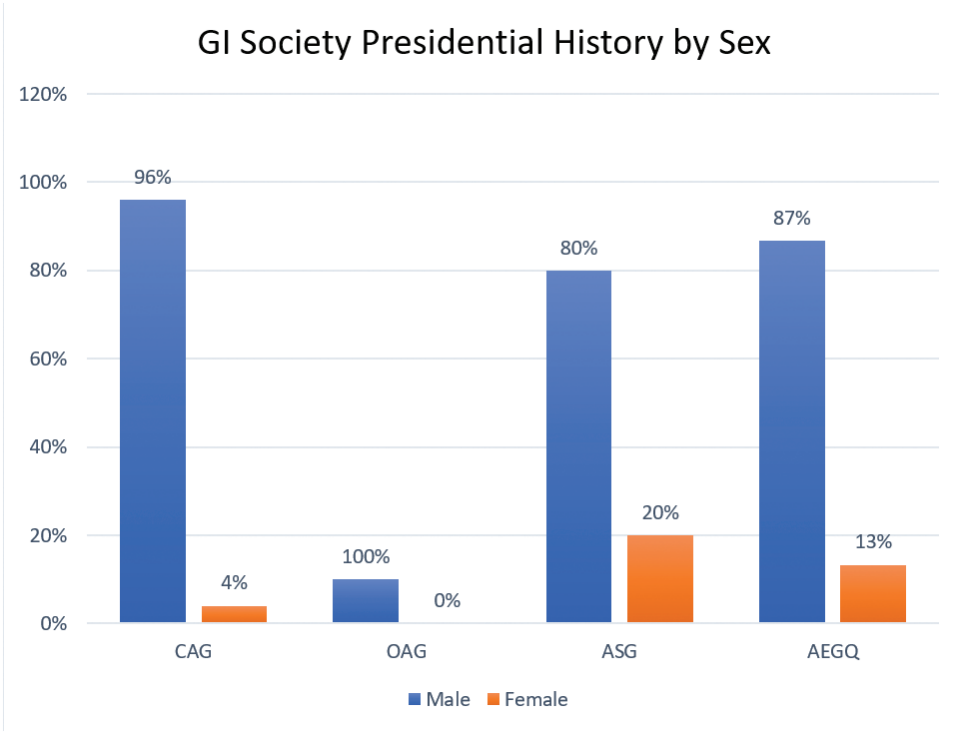
Comparing presidential history by gender across provincial and national gastroenterology associations. AEGQ, Association of Quebec Gastroenterologists; ASG, Alberta Society of Gastroenterology; CAG, Canadian Association of Gastroenterology; OAG, Ontario Association of Gastroenterology.

A review of historical data on past presidents by gender of the four national and provincial GI organizations revealed the following: in CAG’s 60-year history, 2/47 (4.2%) presidents were women. In the OAG’s 22-year history, no (0/9) women presidents had ever been elected. In the AGEQ’s 55-year history, 2/15 (13%) of presidents have been women. In the ASG’s 10-year history, 1/5 (20%) of presidents have been women (Figure 3). The remaining seven provinces and three territories do not have provincial gastroenterology associations.

**Figure 4.**
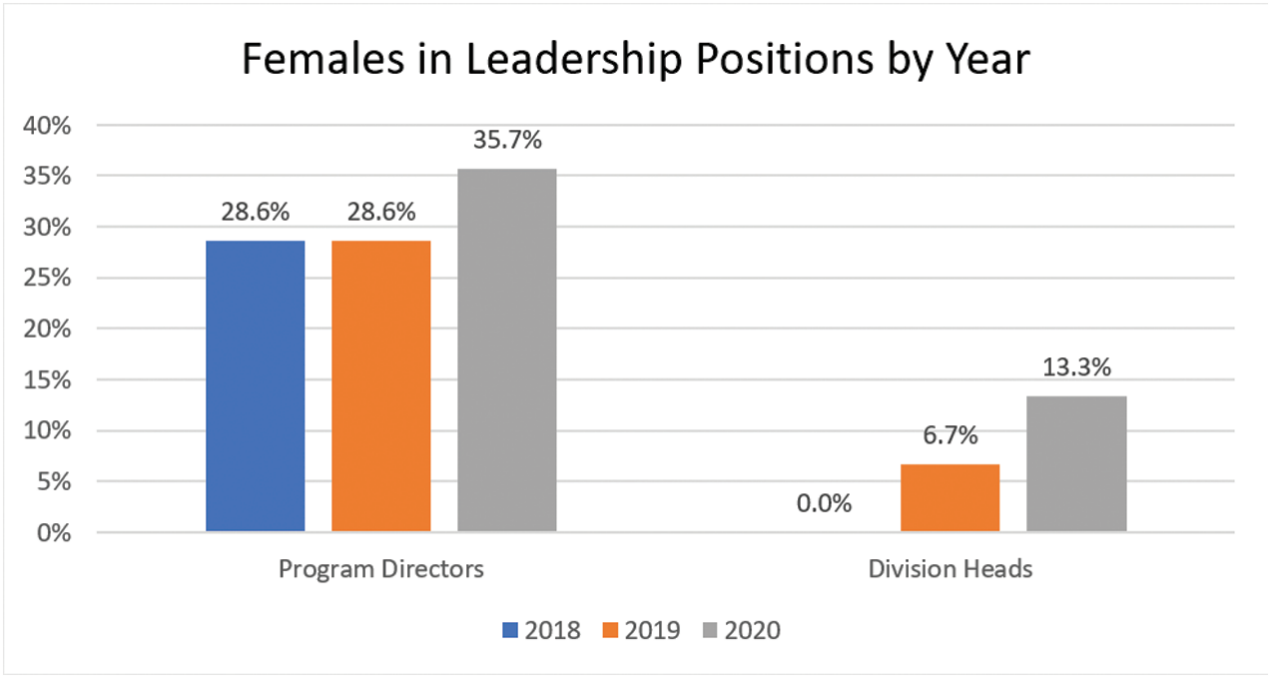
Females in leadership positions as program directors and gastroenterology division heads from 2018 to 2020.

Academic leadership was measured using the proportion of women as university division heads (*n* = 15) and university GI training program directors (*n* = 14) across Canada from 2018 to 2020. National data prior to this were not reliably available. University division heads were 0% women (0/15) in 2018, 6.7% women (1/15) in 2019 and 13.3% women (2/15) in 2020. University GI training program directors were 28.6% women (4/14) in 2018, 28.6% women (4/14) in 2019 and 35.7% women (5/14) in 2020 (Figure 4).

Scholarly leadership was measured using the number of women oral speakers at three provincial and one national GI association annual conferences spanning from 2013 to 2020. Women speakers at the CAG Canadian Digestive Diseases Week (CDDW) symposiums varied 27% to 42% from 2016 to 2020 with an average of 32.7% (86/263). Women speakers at the OAG annual conference varied 13% to 33% from 2015 to 2020 with an average of 23.3% (10/43). Women speakers at AGEQ’s annual conference varied 10% to 35% from 2015 to 2019 with an average of 24.1% (20/83). Women speakers at ASG’s annual conference varied 31% to 43% from 2013 to 2019 with an average of 35% (111/317).

## Discussion

Our study emphasizes that women’s under-representation is still an active issue at all levels of the GI career path. Though there was proportionate representation of women between applicants and those ultimately accepted into internal medicine and GI residency programs respectively, there was a sizable drop in women from 49.5% of overall trainees to 35.3% when moving onto GI residency. Cardiology was similarly disproportionate in women’s representation from applying to matching but had lower numbers overall: only 27.8% matched were women. This study did not delve into potential reasons for the drop off of women applying to gastroenterology residency. It is possible that this decrease in numbers reflects personal choice such as wishing to avoid procedures. However, gender-related factors cannot be discounted completely.

General surgery consistently accepted more women during the study period (54.4% on average) despite having more men apply each year. In fact, general surgery accepted even more women overall and in proportion to menthan did internal medicine. In Lorello et al.’s study of female applicants and matriculants in Canadian residency programs across multiple specialties, they also found this increasing trend of female representation within general surgery (on average 47.2% from 1995 to 2019) to be significant (*P* < 0.001) (8). Although their study did not elicit reasons for this phenomenon directly, other studies have shown potential reasons to include increased female representation within the speciality overall, especially among leadership such as program directors, attracts more female applicants and fostering interest in the specialty early in medical school such as through the use of specialty interest groups can make a difference (9,10). This is encouraging proof that applicant and matched rates for women can be improved even within a male-dominated specialty.

From our statistics, women’s under-representation in GI begins early at the residency training level. The reasons for this gender divide were not explored in this study; however, Heathcote et al. felt a possible combination of institutional culture biases toward females was to blame. In Heathcote’s study, both male and female respondents felt it was more difficult for females to enter GI residency training. This was further compounded by female residents feeling they had different expectations set for them and had greater difficulty in proving themselves to both peers and senior colleagues (6). In 2002, Arlow et al. conducted a survey focusing on gender differences in the selection and training of GI fellows. They noted gender differences even before starting GI training: during the selection process, more female applicants were asked questions in their interviews directly related to their gender compared to male applicants (11). Further study is needed to understand in-depth the inherent gender biases present in the gastroenterology training paradigm, especially in Canada. The comparisons made in this study emphasize that this issue is complex. Based on the IM and GS comparative data, women do choose high workload procedural-based disciplines and that some procedural programs can show a disproportionate female preference even in the match process. However, for unclear reasons, by PGY4, women are under-represented in procedural based, high workload, competitive specialties such as GI and cardiology.

Women in GI did not improve their representation as they became staff and vied for leadership positions. This study showed that leadership roles including presidential positions for provincial and national GI organizations, university division heads and program directors were dominated by men in Canada. As a comparison, American gastroenterology societies also demonstrated gender disparity for females among their board of governors (AASLD 36%, ASGE 32%, AGA 23%, ACG 16%), though their numbers were more proportionate than Canada. In fact, 2017 marked the first time that all four national GI society (AASLD, ASGE, AGA and ACG) presidents were female (12). However, when multiple specialties are compared, there is wide variation. In a cross-sectional study of presidential histories by gender of 43 medical specialty societies in the USA, Silver et al. showed that men served as presidents in 82.6% of years compared to women serving for only 17.4% years (13). Gastroenterology specifically showed only 10% of years being served by a woman president. Woodward et al. analyzed the gender division in academic leadership roles including program director, associate program director and division chief across 163 GI fellowship programs. A higher proportion of males held each position by a large margin: program directors were 82% male; associate program directors were 72% male and division chiefs were 93% male (14). It appears Canada is not alone in having disproportionately low women’s representation among gastroenterology leadership.

The major contributing factor to explaining both the current disparity and potential future improvement was noted by Diamond et al. as the statistically significant longer duration of careers for male gastroenterologists. Males had a median career duration of 20 years compared to just 11 years for their female counterparts. When career duration was accounted for in the analysis, gender differences among publication productivity and academic rank almost disappeared (15). A survey-based study completed in 2019 compared motivations for seeking leadership roles between males and females. Though they also found more males than females in leadership positions (52% versus 36%), they noted that among those who completed their training in the previous 5 years, more women than men held leadership roles (25% versus 6%) (16). This hopefully points to improvements in leadership involvement among newly graduated women gastroenterologists and beyond.

An important measure of academic leadership and productivity is the quality and quantity of research publications. There are several studies in the literature describing gender disparities within GI academic publication authorship. Long et al. analyzed female authorship across five major GI journals over the last 20 years and found mixed results. The percentage of female first authorship had been increasing overtime and remained proportionate to the number of academic female gastroenterologists. However, female senior authorship occurred at a lower rate than expected (5). Looking at GI societal guidelines and technical reviews between 2007 and 2019, Bushyhead et al. found that 18% of first authors were female with 21% of overall authorship being female. Statistically significant improvement in female authorship was only noted for AASLD guidelines (17).

Our study further expanded gender demographic trends in scholarly activity by focusing on oral presentations at national gastroenterology conferences in Canada over the last 5 years. Both the OAG’s and CAG’s proportion of women oral presenters showed an increasing trend and remained proportionate to the number of academic women gastroenterologists. Enestvedt et al. similarly found the proportion of female faculty teaching courses at the American Society for Gastrointestinal Endoscopy’s sponsored programs to steadily be increasing over time (18). A United Kingdom-based study also found female proportionate representation among their national gastroenterology conference attendance and scholarly contribution (19). In this particular area of academia, women GIs in Canada are showing improving trends in research productivity and publications similar to their counterparts in other countries.

This paper has several limitations. Firstly, our paper is mostly across-sectional analysis and offers little insight into temporal trends, thereby preventing us from assessing the progress toward gender equity within GI over a longer period of time. We used a binary definition of gender (men versus women) but understand that this is a simplification which does not account for those that do not define themselves as such. The majority of our data were acquired from publicly available sources and with the cooperation of CaRMS but both have logistical limitations. Given the small applicant pool for the sub-specialty match of GI, CaRMS could not provide information regarding exam scores, scholarly activity, research productivity or the quality of letters of recommendation for individual applicants, in an effort to protect confidentiality. Similarly, we have no information regarding how GI residency training programs choose their successful candidates and the relative importance assigned to these factors. To our knowledge, there is no standardization in this process across programs. Therefore, accounting for such confounding factors could not be undertaken and drawing a definite conclusion about the impact of gender in the post-graduate selection process is not possible. Similarly, for leadership statistics, the gender breakdown of various leader roles was acquired from public websites on which a limited amount of information was available. We could not study individual leader attributes such as their duration of career, clinical or nonclinical status, previous leadership roles or research achievements, potential confounders which could affect an applicant’s success in obtaining a leadership position. It is similarly unclear how organizations choose leaders and there is almost certainly no standardization in this process. Therefore, again it is difficult to ascribe true causality to the impact of gender on leadership success, given the unknown impact of these other important contributors. Lastly, statistics such as how many women gastroenterologists declined leadership positions, how many were not offered leadership opportunities in the first place or the overall denominator of the pool of eligible applicants from which leaders were drawn, are not publicly available. Therefore, it is possible that women’s representation at the leadership levels are not disproportionate, instead they can be considered simply low overall.

Constructive approaches to facilitating women’s engagement in the field of gastroenterology are a critical step forward in the process. As women proportionately match into gastroenterology, just prior to this step can be highlighted as a crucial tipping point where intervention may play a key role. Targeted mentorship of GIM residents to encourage application to a GI residency training program may be helpful and specifically woman–woman mentorship pairs may be particularly effective. Ongoing career mentorship may also prove to be impactful. Early discussion of work–life balance and family planning during residency training and early-career years may assist with strategic planning of life and career milestones. Resource development to prevent and treat burnout, especially for those providers who may be the primary person responsible for multiple competing obligations may be helpful. This is particularly important knowing that women are more often responsible for childcare and other domestic responsibilities (6).

## CONCLUSION

Despite gender equivalency in medical school and internal medicine residency, women are under-represented in GI sub-specialty residency programs similar to other PGY4 entry procedure-based sub-specialties such as cardiology. Women are also under-represented in major academic leadership roles and at the provincial and national organizations’ leadership levels. Scholarly activity such as oral presentations at conferences do however appears to show an improving trend over time. This study adds to the paucity of data available in gender literature related to the early training steps such as residency. This study also highlights the minimal representation of women in leadership positions. There is a clear need for further studies to understand why gender disparities exist at each career step to help define targeted interventions.

## AUTHOR CONTRIBUTIONS

N.J. contributed to the planning and conduction of the study, collection and interpretation of data and drafting of the manuscript. She has approved the final draft submitted. J.L. contributed to the collation, analysis and interpretation of data for the study. She has approved the final draft submitted. N.B. contributed to the planning and conduction of the study, interpretation of the data and critical revisions of the manuscript for important intellectual content. She has approved the final draft submitted. Guarantor of the article: N.B.

## Funding

No funding sources to disclose.

## CONFLICT OF INTEREST

No potential conflicts of interest to declare.
